# Comparison of mesenchymal stem cells obtained by suspended culture of synovium from patients with rheumatoid arthritis and osteoarthritis

**DOI:** 10.1186/s12891-018-1998-6

**Published:** 2018-03-09

**Authors:** Yuji Kohno, Mitsuru Mizuno, Nobutake Ozeki, Hisako Katano, Koji Otabe, Hideyuki Koga, Mikio Matsumoto, Haruka Kaneko, Yuji Takazawa, Ichiro Sekiya

**Affiliations:** 1 Center for Stem Cells and Regenerative Medicine, Tokyo Medical and Dental University, 1-5-45 Yushima, Bunkyo-ku, Tokyo, 113-8510 Japan; 2 Department of Joint Surgery and Sports Medicine, Tokyo Medical and Dental University, 1-5-45 Yushima, Bunkyo-ku, Tokyo, 113-8510 Japan; 3 Department of Orthopaedics, Juntendo University School of Medicine, 3-1-3 Hongo, Bunkyo-ku, Tokyo, 113-8431 Japan

**Keywords:** Synovial mesenchymal stem cell (synovial MSC), MSC mobilization, Suspended synovium culture model, Rheumatoid arthritis, Osteoarthritis, Harvested cell number, Chondrogenic potential

## Abstract

**Background:**

Mobilization of mesenchymal stem cells (MSCs) from the synovium was revealed using a “suspended synovium culture model” of osteoarthritis (OA). The pathology of rheumatoid arthritis (RA) differs from that of OA. We investigated whether mobilization of MSCs from the synovium also occurred in RA, and we compared the properties of synovial MSCs collected from suspended synovium culture models of RA and OA.

**Methods:**

Human synovium was harvested during total knee arthroplasty from the knee joints of patients with RA (*n* = 8) and OA (*n* = 6). The synovium was suspended in a bottle containing culture medium and a culture dish at the bottom. Cells were harvested from the dish and analyzed.

**Results:**

No significant difference was observed between RA and OA in the harvested cell numbers per g of synovium. However, the variation in the number of cells harvested from each donor was greater for RA than for OA. The harvested cells were multipotent and no difference was observed in the cartilage pellet weight between RA and OA. The surface epitopes of the cells in RA and OA were similar to those of MSCs.

**Conclusion:**

Mobilization of MSCs from the synovium was demonstrated using a suspended synovium culture model for RA. The harvested cell numbers, chondrogenic potentials, and surface epitope profiles were comparable between the RA and OA models.

## Background

Mesenchymal stem cells (MSCs) are seldom detected in samples of synovial fluid from the knees of healthy volunteers [1–3]. However, the number of MSCs in the synovial fluid increases with the radiologic grade of osteoarthritis (OA) [2]. The cell morphology and gene profiles of MSCs from the synovial fluid of patients with OA have a stronger resemblance to those from the synovium than from the bone marrow, suggesting an intriguing possibility that MSCs found in synovial fluid originate from the synovium and that OA can trigger the release of MSCs from the synovium into the synovial fluid [1]. We have recently demonstrated a “suspended synovium culture model” in which MSCs from the synovium of patients with OA were mobilized into a non-contacted culture dish through culture medium [4].

MSCs are also found in the synovial fluid of patients with rheumatoid arthritis (RA), an autoimmune condition characterized by inflammation, usually in bilateral joints, and systemic features such as fatigue or fever [5–7]. However, an unanswered question is whether the synovium of patients with RA directly releases MSCs into the synovial fluid, as occurs in patients with OA, because the pathological conditions of RA and OA are different. A further possibility is that the use of anti-inflammatory drugs in patients with RA may affect the synovium as a source of MSCs in the synovial fluid.

The primary aim of this study was to evaluate the possible release of MSCs from the synovium in a suspended synovium culture model of RA. Since some properties of synovial MSCs may vary depending on disease etiology, a secondary aim was to compare the properties of synovial MSCs obtained from the suspended synovium culture models of RA and OA.

## Methods

### Synovium harvest and ‘suspended synovium culture’

This study was approved by local institutional review boards (the Medical Research Ethics Committee of Tokyo Medical and Dental University and the Hospital Ethics Committee of Juntendo University Hospital), and informed consent was obtained from all study subjects. Human synovium was harvested during total knee arthroplasty from knee joints of patients with RA (8 donors) and OA (6 donors). The patients ranged in age from 49 to 79 years for RA donors and from 55 to 78 years for OA donors. Patient demographics are listed in Table 1.

**Table 1:** Patient demographics

Group	Patient number	Age	Sex	CRP (mg/dl)	ESR (mm)	Medicine		
						PSL (mg /day)	DMARDs other than biologics	Biologics
RA	#1	49	F	0.1	17	2	MTX	ETN
	#2	79	F	5.7	98			
	#3	65	F	0.02	39	5	TCR, IGU	TCZ
	#4	55	F	2.3	47	2	MTX, SASP	TCZ
	#5	79	F	0.1	12	2.5	BUC, SASP	
	#6	38	F	0.0		2	MTX	TCZ
	#7	50	F	2.5	52		MTX, AZA	
	#8	73	F	0.3	2	2		ETN
OA	#9	62	M	0.1	5			
	#10	72	F	0.1	5			
	#11	78	F	0.0	19			
	#12	72	M	0.0	8			
	#13	55	F	0.2	20			
	#14	73	F	0.1	15			

The mean of age was 61 years old in RA group, and 69 years old in OA group: no significant difference between them (*p* = 0.56 by Mann-Whitney’s U test). *PSL* Prednisolone, *MTX* Methotrexate, *TCR* Tacrolimus, *IGU* Iguratimod, *SASP* Salazosulfapyridine, *BUC* Bucillamine, *AZA* Azathioprine, *ETN* Etanercept, *TCZ* Tocilizumab

**Fig. 1:**
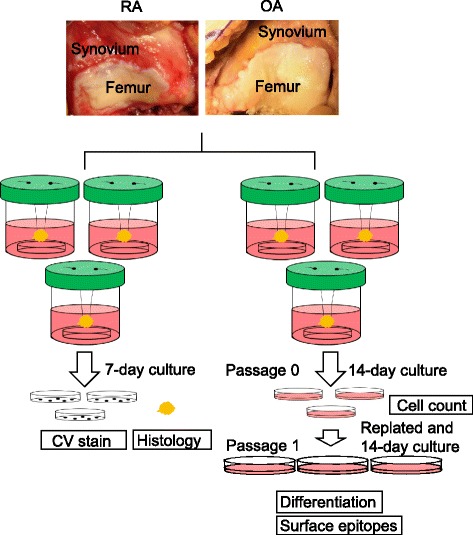
Suspended synovium culture protocol. Human synovium was harvested during total knee arthroplasty from knee joints of patients with rheumatoid arthritis (RA; *n* = 8) and osteoarthritis (OA; *n* = 6). Approximately 1 g of synovium from each donor was suspended in each of six bottles that contained culture medium and a culture dish placed at the bottom of the bottle. After seven days of suspended synovium culture, three dishes were stained with 0.5% crystal violet and the suspended synovium was also examined histologically. After fourteen days of suspended synovium culture, the harvested cell numbers were evaluated from the remaining three dishes for each donor. The cells were passaged again and used for differentiation assays and analysis of surface epitope expression

The synovium was cut into six approximately 1 g pieces with a surgical knife and washed thoroughly with phosphate-buffered saline (PBS; Invitrogen, Carlsbad, CA) to remove blood traces. Each synovium piece was sutured with 4–0 nylon thread and suspended in a 100 mL bottle (Sarstedt, Numbrecht, Germany) containing a 35-mm-diameter culture dish (Thermo Fisher Scientific, Yokohama, Japan) placed at the bottom. The culture dish contained 40 mL of a-modified Eagle medium (a-MEM; Invitrogen) with 10% fetal bovine serum (Invitrogen), and 1% penicillin/streptomycin (Invitrogen) (Fig. 1).

After culturing for 7 days at 37 °C, three dishes were stained with 0.5% crystal violet (Wako, Osaka, Japan) in 10% formalin for 5 min, washed twice with distilled water, and the cell colonies were observed by light microscopy. The synovium before and 7 days after suspended culture was examined histologically.

The other three dishes from each donor were left in the bottles with the suspended synovium for 14 days, and passage 0 cells were collected for cell counting. The cells were then replated, cultured for a further14 days, and analyzed for differentiation and surface epitopes.

### Histological analysis

The synovium before and 7 days after suspended culture was fixed in 10% formalin, embedded in paraffin wax, sectioned at 5 μm, and stained with hematoxylin and eosin. Each sample was assigned to one of three grades according to the thickness of the synovial intima: grade 1 = synovial intima less than four cells thick; grade 2 = synovial intima four to six cells thick; and grade 3 = synovial intima seven or more cells thick [8, 9].

### Differentiation assay

For chondrogenesis, 2.5 × 10^5^ cells were transferred to a 15 ml tube (BD Falcon) and cultured in chondrogenic induction medium containing 10 ng/ml transforming growth factor-β3 (Miltenyi Biotec Japan, Tokyo, Japan) and 500 ng/ml bone morphogenetic protein 2 (BMP-2, Infuse; Medtronic, Minneapolis, MN); this medium was changed every 3–4 days. After 21 days, the cell pellets were embedded, sectioned and stained with safranin O and fast green (Wako, Tokyo, Japan).

Calcification was studied by plating 100 cells in a 60 cm^2^ dish and culturing for 14 days in α-MEM with 10% fetal bovine serum. Adherent cells were subsequently cultured in an osteogenic induction medium containing 50 μg/ml ascorbic acid 2-phosphate (Wako), 10 nM dexamethasone (Wako), and 10 mM β-glycerophosphate (Sigma-Aldrich); this medium was changed every 3–4 days. After 14 days, calcification was assessed by alizarin red (Merck Millipore, Billerica, MA) staining.

Adipogenesis was evaluated by plating 100 cells in a 60 cm^2^ dish and culturing for 14 days to allow colony formation. Adherent cells were cultured in an adipogenic induction medium supplemented with 100 nM dexamethasone, 0.5 mM isobutylmethylxanthine (Sigma-Aldrich), and 50 mM indomethacin (Wako) for an additional 14 days; this medium was changed every 3–4 days. Adipocyte colonies were stained with oil red O (Muto Pure Chemicals, Tokyo, Japan).

### Flow cytometry analysis

Passage 2 cells were suspended in Hank’s Balanced Salt Solution (HBSS) at a density of 5 × 10^5^ cells/mL and stained for 30 min on ice with the following antibodies: CD11b-PE, CD11c-PE-Cy7, CD14-APC, CD31-FITC, CD44-APCH7, CD45-FITC, CD73-BV421, CD90-PE, CD105-PerCP-Cy5.5, CD206-FITC, and HLADR-APC (BD, Franklin Lakes, NJ). Cell surface antigens were analyzed using a triple-laser FACS Verse™ system (BD).

### Statistical analysis

The results were analyzed using Mann-Whitney’s *U* test with GraphPad Prism 6 (GraphPad Software, La Jolla, CA, USA). *P* values < 0.05 were considered significant.

## Results

**Fig. 2:**
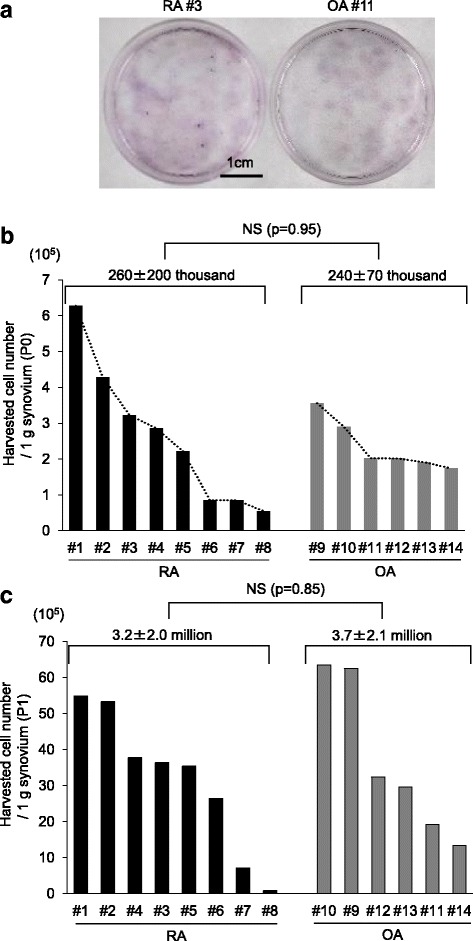
Cell colonies and harvested cell numbers after suspended culture of synovium from patients with rheumatoid arthritis (RA; *n* = 8) and osteoarthritis (OA; *n* = 6). **a** Representative cell colonies stained with crystal violet after 7 days of suspended synovium culture. **b** Passage 0 cell numbers/g synovium after 14 days of suspended synovium culture. **c** Passage 1 cell numbers/g synovium after14 days of culture of passage 0 cells. Average values with standard deviation are shown (RA, *n* = 8; OA, *n* = 6). NS: not significant

After seven days of suspended synovium culture, cell colonies were observed in the dishes in both the RA and OA samples (Fig. 2a). No significant difference was noted for the passage 0 cell numbers between the RA and OA cultures: the passage 0 harvested cell numbers after 14 days of suspended synovium culture was 2.6 × 10^5^ ± 2.0 × 10^5^ cells/g synovium for the RA and 2.4 × 10^5^ ± 0.7 × 10^5^ cells/g synovium for the OA samples (Fig. 2b). However, the passage 0 cell numbers varied greatly among the RA samples depending on the donor, whereas these numbers were similar in the OA samples. An F-test analysis revealed a significant difference in this variation (*P* = 0.04) (Fig. 2b). The harvested cell numbers for passage 1 were 3.2 × 10^6^ ± 2.0 × 10^6^ cells/g synovium for the RA and 3.7 × 10^6^ ± 2.1 × 10^6^ cells/g synovium for the OA samples (Fig. 2c); this difference was not statistically significant (*P* > 0.05).

**Fig. 3:**
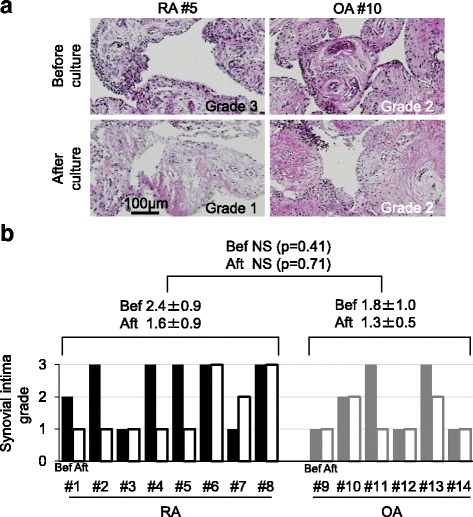
Histological analysis of synovium from patients with rheumatoid arthritis (RA; *n* = 8) and osteoarthritis (OA; *n* = 6) before and after 7 days of suspended culture. **a** Representative sections stained with hematoxylin and eosin. Each synovium was assigned to one of three grades according to the thickness of the synovial intima: grade 1 = synovial intima less than four cells thick; grade 2 = synovial intima four to six cells thick; and grade 3 = synovial intima seven or more cells thick. **b** Synovial intima grade before and after 7 days of suspended synovium culture. Bef: before, Aft: after, NS: not significant

Histological analysis of the synovium before and after 7 days of suspended culture was conducted after assigning each synovium to one of three grades according to the number of cells in the synovial intima (Fig. 3a). The synovial intima grade decreased after suspended culture in four RA donors, remained constant in three RA donors, and increased in one RA donor, whereas it decreased in two OA donors and remained constant in four OA donors (Fig. 3b).

**Fig. 4:**
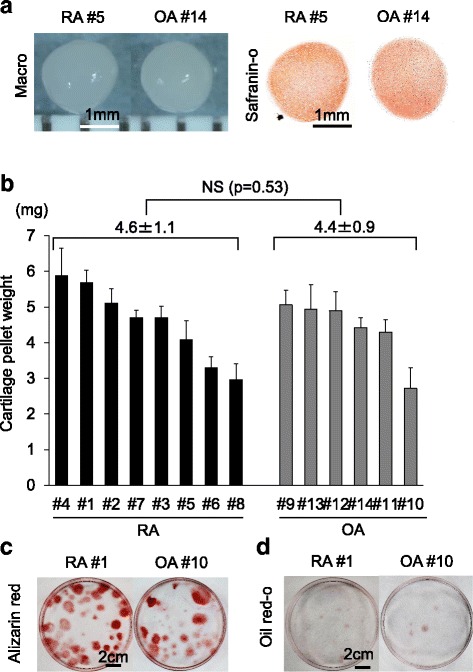
Differentiation assays of the cells passaged after suspended culture of synovium from patients with rheumatoid arthritis (RA; *n* = 8) and osteoarthritis (OA; *n* = 6). **a** Chondrogenesis. Representative macro pictures and histological sections stained with safranin O are shown. **b** Cartilage pellet weight. Average values with standard deviation are shown. NS: not significant. **c** Calcification. Representative cell colonies stained with alizarin red are shown. **d** Adipogenesis. Representative cell colonies stained with oil red O are shown

Differentiation assays confirmed that passage 1 cells formed cartilage pellets that positively stained with safranin O (Fig. 4a) when cultured in chondrogenic medium. The cartilage pellet weight was 4.6 ± 1.1 mg for RA cultures and 4.4 ± 0.9 mg for OA cultures, and this difference was not statistically significant (*P* > 0.05) (Fig. 4b). Passage 1 cells calcified (Fig. 4c) and differentiated into adipocytes (Fig. 4d) when cultured in differentiation media.

**Fig. 5:**
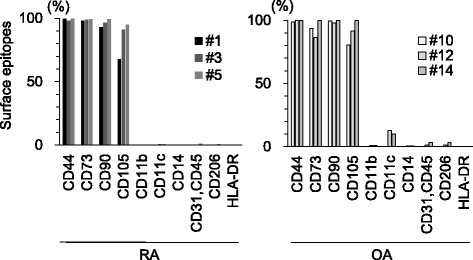
Cell surface markers expressed by synovial cells passaged after suspended culture of synovium from patients with rheumatoid arthritis (RA; *n* = 8) and osteoarthritis (OA; *n* = 6)

The surface epitopes expressed by passage 1 cells included the MSC markers CD44, CD73, and CD90 at high level (> 90%) and CD105 at a moderate or high level (> 60%) (Fig. 5). Passage 1 cells also expressed the hematopoietic markers CD11b, CD11c, CD14, CD31 & 45, CD206 and HLA-DR at low levels (< 10%). The expression profiles appeared similar between the RA and OA cells.

## Discussion

MSCs are characterized by their colony-forming ability and their multipotency for differentiating in vitro into chondrocytes, adipocytes, and osteoblasts [10]. In this study, suspended synovium culture resulted in the appearance of colony forming cells in the dish at the base of the culture bottle. These cells showed characteristics of MSCs, including the potential for multilineage differentiation and expression of surface epitopes common to MSCs. The colony forming cells obtained from the suspended synovium culture therefore appeared to be MSCs.

These MSCs, when derived from the synovium from RA donors, mobilized through culture medium to a culture dish. Marinova-Mutafchieva et al. detected MSCs in the synovial fluid of patients with RA [7]. Yan et al. demonstrated that intraarticular injection of MSCs improved cartilage condition in a murine collagen-induced arthritis model [11]. Taken together, these findings indicate that MSCs are mobilized from the synovium into the synovial fluid and that they participate in tissue repair by ensuring replacement of the mature cells that are lost during the course of disease in RA patients.

The MSCs obtained from the suspended synovium cultures from RA and OA donors had similar mean harvested cell numbers at passage 0 and passage 1, chondrogenic potential, and surface epitope profiles. We previously reported similar results for a comparison of the properties of synovial MSCs obtained using digested synovial cells from RA and OA donors in a standard cell culture [12]. This similarity between the current findings from suspended synovium culture and our previous results from standard cell culture is interesting because migrated cells from synovium and digested cells in synovium are seemed to have similar properties.

The number of cells harvested following suspended synovium culture at passage 0 varied greatly among the RA samples from different donors. We noted a similar response in our previous study that used standard cell culture and determined that the variations were due to differences in nucleated cell numbers per synovium weight between samples obtained from RA and OA donors [12]. The present finding was also due to a wider variation in the nucleated cell numbers per synovium weight among the RA samples when compared to the OA samples.

The changes in the synovial intima grade (Fig. 3) and the harvested cell number (Fig. 2) were not correlated. We had expected that the synovial intima grade would decrease following suspended culture because the synovial intima cells moved through the medium from the suspended synovium into the dish. Two reasons may explain why the synovial intima grade did not decrease in all donors. One reason may be that the same area of the synovium could not be evaluated histologically before and after the suspension. A second reason may be that both synovial intima cells and subsynovial cells may have moved through the medium into the dish.

We previously demonstrated a relationship between the synovitis score and the total yield of synovial MSCs in a rat carrageenan-induced arthritis model [13]. In the current human study, C-reactive protein (CRP) levels were over 2 mg/dl, and erythrocyte sedimentation rate (ESR) levels were over 40 mm in RA donors #2, #4, and #7, whereas these levels were within the normal limits in all patients with OA (Table 1). In only RA #7 were the harvested cell numbers of both P0 and P1 lower (Fig. 2b, c) and the synovial intima grade determined to be lower by histological analysis (Fig. 3). Therefore, serological inflammation levels did not affect the harvested cell number after suspended synovium culture, and these levels did not seem to affect the synovial intima grade determined by histological analysis. Drugs used to treat RA might affect these outcomes.

In the current study, synovium was suspended in the culture medium containing FBS. This raised the question whether MSCs would also mobilize from the synovium if synovial fluid from the patients was used instead of culture medium. We previously found a greater expansion of synovial MSCs derived from OA donors when the MSCs were cultured in a two-dimensional system containing autologous synovial fluid than in a culture medium containing FBS [14]. This suggests that synovial MSCs would also mobilize into synovial fluid in the suspended synovium culture model.

In this study, mobilization of MSCs from the synovium was demonstrated by the “suspended synovium culture model.” We observed a direct migration of the cells from the synovium to a dish placed on the bottom of the culture bottle using time-lapse video (data not shown). The movie showed that many cells were released from the synovium just after the culture started, the cells migrated to the dish, and some of the cells formed cell colonies.

Inherent synovial fibroblasts play an important role, especially in RA. In the suspended culture model, two possibilities arose: the mobilized cells could be exclusively MSCs or they could be a mixture of MSCs and fibroblasts. The time-lapse movie indicated that the suspended synovium releases both fibroblasts and synovial MSCs, and that after 14 days of culture, most of the cells are MSCs because MSCs are remarkably different from fibroblasts in terms of their high proliferative potential and colony forming ability.

We did not show unstimulated cells as controls for the differentiation assays. We have previously reported that MSCs do not differentiate into chondrocytes unless cultured in a chondrogenic medium [15, 16]. The MSCs also do not differentiate into adipocytes without an adipogenic medium [17]. Many papers have reported that MSCs are not calcified without a calcification medium [18]. Therefore, we did not prepare unstimulated cells as controls for the differentiation assays.

In this paper, we used the term “calcification” rather than “osteogenesis.” We had previously investigated gene expression profiles of MSCs during chondrogenesis, adipogenesis, and “osteogenesis.” The expression of chondrogenesis-related genes, such as Sox9, Aggrecan, and COL2A1, increased during chondrogenesis [19], as did the expression of adipogenesis-related genes, such as PPAR*γ*, LPL, and FABP4, during adipogenesis [17]. However, we were unable to confirm a significant increase in the expression of osteogenesis-specific genes, such as Osterix, Runx2, and Osteocalcin, during “osteogenesis,” despite the positive staining of the MSCs with alizarin red (data not shown). For that reason, we have used the term “calcification” instead of “osteogenesis” [1, 3, 12].

We propose three limitations for the model and this study. One limitation was the small sample number, which precluded the performance of detailed analyses. The second limitation was the varied treatment histories of the patients with RA, which precluded a full analysis of the effects of drugs. The third limitation was the low yield of passage 0 synovial MSCs, which prevented analysis of differentiation and surface epitope expression. One additional passage of passage 1 synovial MSCs may reduce the differences observed in the properties of synovial MSCs obtained from RA and OA donors.

## Conclusion

We revealed a mobilization of MSCs from suspended synovium from a RA donor through culture medium into a culture dish. The harvested cell numbers at passage 0 showed a greater variation for RA samples than for OA samples. The mean harvested cell numbers at passage 0 and 1, chondrogenic potential, and MSC surface epitope expression were similar for synovium from RA and OA donors.

## Abbreviations

BMP-2: Bone morphogenetic protein 2
CRP: C-reactive protein
ESR: Erythrocyte sedimentation rate
MSCs: Mesenchymal stem cells
OA: Osteoarthritis
RA: Rheumatoid arthritis
α-MEM: Alpha minimum essential medium
